# A Codeword-Independent Localization Technique for Reconfigurable Intelligent Surface Enhanced Environments Using Adversarial Learning

**DOI:** 10.3390/s23020984

**Published:** 2023-01-14

**Authors:** Xuanshu Luo, Nirvana Meratnia

**Affiliations:** Department of Mathematics and Computer Science, Eindhoven University of Technology, 5600 MB Eindhoven, The Netherlands

**Keywords:** localization, reconfigurable intelligent surface (RIS), representation learning, domain generalization, domain adversarial neural network (DANN)

## Abstract

Reconfigurable Intelligent Surfaces (RISs) not only enable software-defined radio in modern wireless communication networks but also have the potential to be utilized for localization. Most previous works used channel matrices to calculate locations, requiring extensive field measurements, which leads to rapidly growing complexity. Although a few studies have designed fingerprint-based systems, they are only feasible under an unrealistic assumption that the RIS will be deployed only for localization purposes. Additionally, all these methods utilize RIS codewords for location inference, inducing considerable communication burdens. In this paper, we propose a new localization technique for RIS-enhanced environments that does not require RIS codewords for online location inference. Our proposed approach extracts codeword-independent representations of fingerprints using a domain adversarial neural network. We evaluated our solution using the DeepMIMO dataset. Due to the lack of results from other studies, for fair comparisons, we define oracle and baseline cases, which are the theoretical upper and lower bounds of our system, respectively. In all experiments, our proposed solution performed much more similarly to the oracle cases than the baseline cases, demonstrating the effectiveness and robustness of our method.

## 1. Introduction

Modern wireless communication systems are expected to offer not only better quality of service (QoS) but also software-defined radio for diverse ubiquitous applications [1]. To realize these expectations, (beyond) fifth-generation (5G) mobile telecommunication systems consider the reconfigurable intelligent surface (RIS) as a crucial component for their capability to customize radio frequency (RF) propagation properties, enabling configurable wireless networks [2,3,4]. Specifically, the RIS contains a two-dimensional array of discrete elements, whose electromagnetic impedance are individually tunable [5,6]. This theoretically supports artificial manipulation of signal reflection, diffraction, refraction, polarization, and absorption [7,8,9].

Leveraging higher-frequency RF for faster data rates is generally accepted for modern communication networks [10,11], whereas it results in more severe signal attenuation when there is no line-of-sight (LoS) link between the base station (BS) and the device of interest (DOI) due to weaker diffraction abilities [12,13]. Therefore, among the mentioned RIS functionalities, controlling the signal reflection properties is the most influential one, since it prevents excessive received signal strength indicator (RSSI) drop by constructing an RIS-assisted link to reflect the incoming signal to the direction towards the DOI in non-line-of-sight (NLoS) areas [14,15]. RISs achieve this by assigning suitable complex-valued (CV) RIS codewords to specify the beamforming configurations of all RIS elements [16,17].

Such enhancement particularly promotes research on the localization problems in RIS-enhanced environments. Conventional localization techniques in wireless communication networks utilize time of flight (ToF) [18] and RSSI [19], among other things, to obtain high-accuracy location estimation in different scenarios [20]. However, these methods cannot be directly adopted in RIS-enhanced environments, since the RIS changes original signal propagation properties, inducing wrong inferences of distances or locations.

Some related studies proposed sophisticated communication models and utilized 3D geometric information to either directly calculate DOI positions or analyze estimation error bounds [16,21,22]. However, all these works require detailed CV channel gains/matrices among BSs and DOIs based on accurate field measurements (e.g., ray tracing and wave optics [8]), which are hardly available in practice. Meanwhile, since multiple-input, multiple-output (MIMO) [23] orthogonal frequency-division multiplexing (OFDM) [24] is an accepted paradigm for modern wireless networks [25], as the numbers of BSs and DOIs increase, these models have to consider more and more data links on all sub-carriers between devices. Hence, the fast-growing complexity of these methods leads to long inference time [26], which severely limits their usability in real-world deployments. Considering these drawbacks, other solutions measure the RSSI from known BSs for data-driven fingerprint-based localization [27,28,29,30] in RIS-enhanced environments [31,32,33], as the fingerprints are much easier to collect than field measurements [34] and are far simpler than CV channel matrices [33,35]. However, these methods usually involve cooperation with optimized RIS codewords to provide high-accuracy localization, which unrealistically assumes that the RIS is solely deployed for localization rather than more pressing demands in modern high-frequency wireless networks, such as enlarging the signal coverage and increasing the overall network throughput [8,32]. These approaches also share a common disadvantage in that the CV RIS codewords are indispensable during location inference, which means there are huge additional communication burdens when transmitting large CV vectors to DOIs.

In RIS-enhanced environments, the fingerprints are affected by not only locations but also codewords. The RIS codewords may change at any time, and as such, the dataset containing RSSI measurements used for offline training is unlikely to contain fingerprints corresponding to all possible codewords. Then, during online inference, the localization system will possibly make inferences using fingerprints of unknown codewords, leading to large estimation errors, because the location estimator only fits the codeword distribution of the training dataset. In the field of statistical learning, such performance degradation caused by differently distributed training and test data is known as a *domain generalization* (DG) problem [36], where the domain here contains all possible RIS codewords.

Fortunately, as codewords directly control signal propagation in RIS-enhanced environments, the impact of codewords on fingerprints is not irregular. This observation inspired us to learn from this correlation and try to decouple the dependency of fingerprints on codewords to obtain codeword-independent representations of fingerprints for localization in RIS-enhanced environments. Such a system will only be able to generate fingerprint representations highly related to locations. By doing so, we can (i) solve the RIS codeword domain generalization problem and avoid large estimation errors for fingerprints of unknown codewords, and (ii) predict the locations without the corresponding codewords during online inference.

We realized this idea using adversarial learning applied on the codeword domain using a domain-adversarial neural network (DANN) [37] framework consisting of three parts, i.e., a fingerprint feature extractor, a location estimator, and a codeword discriminator. Specifically, during the offline training stage, the feature extractor and the location estimator together behave like conventional fingerprint-based localization systems, i.e., minimizing the location-estimation error. Simultaneously, the codeword discriminator performs adversarial learning on codewords by reversal gradients during backpropagation to guide the feature extractor to generate codeword-independent representations. Our main contributions are as follows:

1. We analyze the localization problem in RIS-enhanced networks in depth and propose a novel paradigm without additional assumptions on the RIS codewords, which also supports online inference without codewords.
2. We propose a localization solution based on codeword-independent representation learning using the domain-adversarial neural network framework to solve the DG problem.
3. Our proposed solution is extensively evaluated using the DeepMIMO dataset [38]. We designed oracle and baseline cases for comparison, which convincingly demonstrate that our solution achieves accurate localization even for unknown RIS codewords. Additional experiments on the system parameters further demonstrate the rationality and robustness of the proposed solution.

## 2. Related Work

### 2.1. Localization in RIS-Enhanced Environments

Most research on localization in RIS-enhanced environments is model-driven. Model-driven localization methods usually have specific optimization goals to directly or indirectly facilitate localization. Wymeersch et al. [39] utilized Fisher information analysis [40,41] to select the best RIS codewords for localization. Elzanaty et al. [21] designed an RIS codeword optimization scheme to maximize the signal-to-noise ratio at the DOI to facilitate localization, and provided analysis on the Cramér–Rao lower bound [42] of the localization error. These works, however, are only feasible when the channel gains/matrices among BSs, DOIs, and the RIS are known, which requires accurate field measurements (e.g., ray tracing [8]), thereby greatly restricting their applicability. Meanwhile, modern communication networks usually adopt MIMO antennas using OFDM. Consequently, as the numbers of BSs and DOIs increase, the complexity of these methods increases fast. Hence, many studies explicitly restrict the number of devices in the environment. For instance, in the analysis of Wymeersch et al. [39], the number of BSs was set to one for simplicity. The fast-growing complexity of location calculation induces unacceptable time delay during location inference when there is a vast number of requests for localization services.

Only a few works noticed the mentioned drawbacks, utilized fingerprints, and proposed data-driven methods. Zhang et al. [35] designed a codeword selection method that aims to enlarge the differences in fingerprints in adjacent positions for high-accuracy localization. They further refined this work and proposed an integrated localization system in [31], realizing centimeter-level accuracy. Huang et al. [33] also picked the best available RIS codeword, but simply for signal strength improvement, which indirectly enhances localization performance. However, the prerequisite for these methods is the RIS is deployed solely for localization, rather than more important tasks such as enlarging the signal coverage and increasing the network throughput [32]. Such assumptions are very unlikely to be met in real-world deployments. Even if realized, these methods all require RIS codewords for location inference, which results in inescapable additional communication burdens on the networks to continuously transmit large CV vectors, as requested by the DOIs. As the number of DOIs grows, the whole localization system would eventually collapse and affect the normal operations of RIS-enhanced communication systems.

### 2.2. Domain Generalization

Domain generalization (DG) problems [36] usually occur in deployments of statistical learning techniques due to distinct distributions of offline training data and online data. This phenomenon is very common because collecting a training dataset perfectly representing real-world scenarios tends to be impossible. Although statistical learning methods, especially for DNNs, have recognized generalization capabilities, this is only true for online data with the same distribution as the training data [43].

There are many research topics addressing the DG problem. Multi-task [44] learning reuses the data representations for training on different related tasks to help the model to perform better for the original task. However, multi-task learning models only work for the domains they have already seen, which hardly fully solves the DG problem. Transfer learning [45,46] pre-trains the models using data from classes with numerous samples, then fine-tunes the model’s parameters with data from other classes. This canonical pipeline can be used for solving the DG problem by fine-tuning the models using data from different domains. However, transfer learning methods require data from other domains for training, which is not always available [47]. Meta-learning techniques [48,49] recently became popular as promising solutions to DG problems. Meta-learning tries to learn a universal rule from various datasets and various tasks, i.e., learning to learn [50], which is close to human learning habits. Although meta-learning methods are attractive for DG problems, their computational time is always extremely long [51]. Meanwhile, their DG performances are sometimes unsatisfactory due to incidental overfitting on training datasets [51]. Data augmentation [52] aims to generate more samples for training for greater data diversity according to the existing training dataset to fulfill DG. However, in our problem, we cannot create meaningful fingerprints without channel matrices.

Representation learning [53] aims to unify either the data representations or the inference results, or both training and inference for different domains [36,54]. When extracted data representations are invariant for different domains and only relevant to the concerned task, domain generalization is achieved. In this context, DANN [37,55] is a straightforward solution involving adversarial learning on domain labels to generate data representations that are irrelevant to the concerned domain [56,57]. DANN implements this by reversing the gradient of the domain discriminator during backpropagation [58].

## 3. Preliminaries

In this section, we first provide fundamental information about RISs, including their working principles and their respective methods for calculating path loss in RIS-enhanced environments. Next, we review our prior work describing the way to transform fingerprints into graphs. Finally, the training process of DANN frameworks is elaborated.

### 3.1. Reconfigurable Intelligent Surfaces

**Figure 1:**
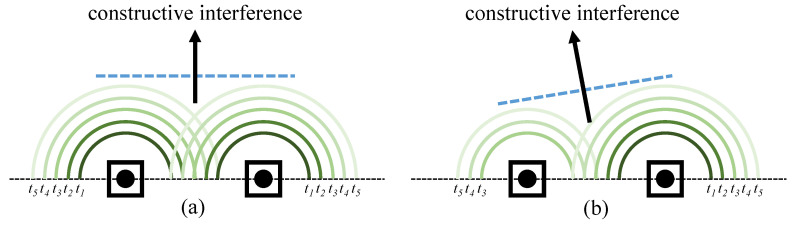
Different beamforming directions for two radiators with different time delays. In (**a**), two radiators emit the same signal at the same time. In (**b**), two radiators also emit the same signal, but the left one starts from $t_{3}$, inducing a different constructive interference compared with (**a**), thereby a left direction signal.

In communication systems equipped with MIMO antennas, the directions of beamforming depend on the constructive interference of all RF signals from radiators [59]. Figure 1 illustrates that the signal directions of two scenarios are different due to different time delays of two radiators. Such time delays are manifested as phase shifts in the frequency domain. Hence, the two radiators together in Figure 1a,b can be considered as a phase shifter.

**Figure 2:**
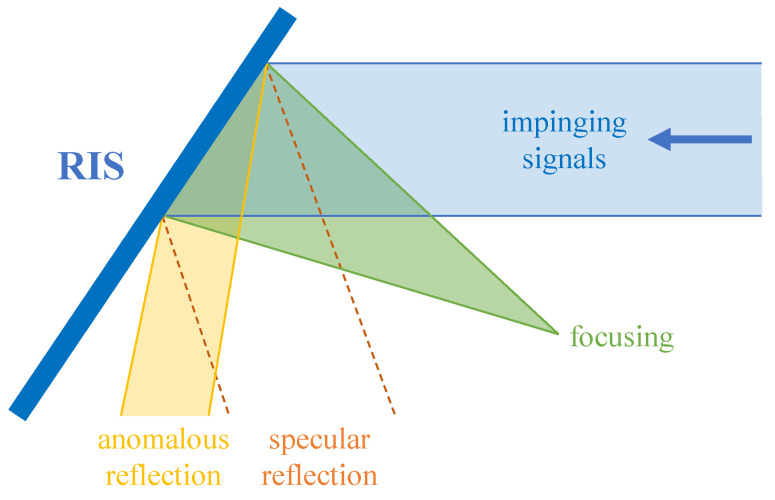
RIS behaviors: anomalous reflection (including specular reflection) and focusing.

RISs are capable of reflecting the incident signals in the desired directions, as an RIS contains a 2D array of such phase shifters whose phases are individually reconfigurable and controlled by assigned impedance [5]. Codewords are CV vectors characterizing phase shifts of all RIS elements. Given an RIS with *M* elements, its codeword $\psi \in C^{M\times 1}$, where for the element *m* with a phase shift ϕ, $\psi_{m}=e^{j\phi }$ [60]. Codewords determine the RIS behaviors, which generally include two categories: anomalous reflection and focusing [61,62], as shown in Figure 2. Concretely, anomalous reflection means that the RIS reflects the impinging signals towards arbitrary directions in parallel, thereby also including the common specular reflection (the angle of incidence equals the angle of reflection). Focusing would converge the reflected signals to one point, which is very effective for a single DOI, but at the same time means low versatility. Hence, in this paper, we only consider the RIS as a pure anomalous reflector, whose function is merely the manipulation of phase shifts.

**Figure 3:**
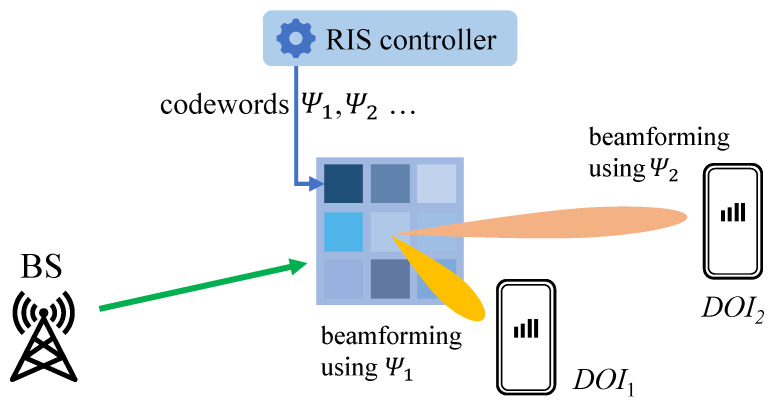
Different beamforming directions given different codewords.

In this context, assigning different codewords to the RIS can help obtain different reflection angles for different devices/functions, as shown in Figure 3. Hence, when the codewords change, shifts in RSSI values occur even for the same locations, which consequently downgrade the estimation accuracy of conventional RSSI fingerprint-based localization solutions in RIS-enhanced wireless communication systems.

**Figure 4:**
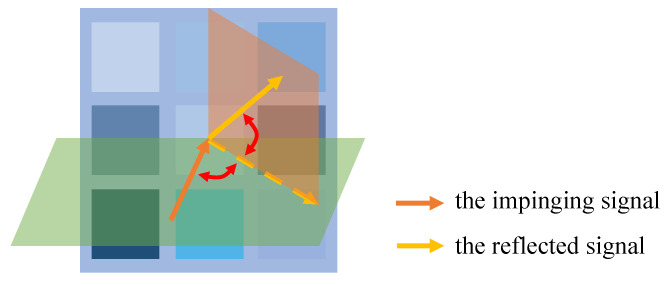
A sketch map illustrating an RIS manipulating reflection angles in two dimensions.

Limited by material and production costs, the number of codewords supported by the RIS is usually restricted [63]. The number of available RIS codewords *C* depends on the RIS’s resolution [64]. For instance, if an RIS supports a phase shift range of 180° with a 30° resolution, then $C={\left(180/30\right)}^{2}=36$, because the RIS can manipulate reflection angles in two planes, as shown in Figure 4. An alternative way to denote the resolution is the number of bits; i.e., a resolution of *b* bits means $C=2^{b}$ [65]. Most current RIS prototypes support only 1 bit (C=2) or 2 bits (C=4) [66,67], and a few achieve up to 6 bits (C=64) [68].

#### 3.1.1. RSSI Calculation in RIS-Enhanced Environments

The DeepMIMO dataset [38] for evaluation in this paper does not provide RSSI values, thereby we cannot directly build fingerprints for experiments. Hence, this section elaborates on the RSSI calculation method in RIS-enhanced environments using channel matrices among BSs, DOI, and the RIS given by the DeepMIMO.

To calculate the RSSI measured by DOI, a common practice is to apply a link budget model, which describes all power gains and losses during the whole signal transmission process [69]. In general, the power gains (dB) $G_{p}$ come from transmitter output power and transmitter/receiver antenna gain, whereas the power losses mainly include transmitter/receiver losses (dB) $L_{p}$ and path loss (dB) *PL* [70]. Then, applying a similar link budget model in [71], we have

$$\mathrm{RSSI}\left(\mathrm{dB}\right)=G_{p}-L_{p}-\mathrm{PL}.$$

**Figure 5:**
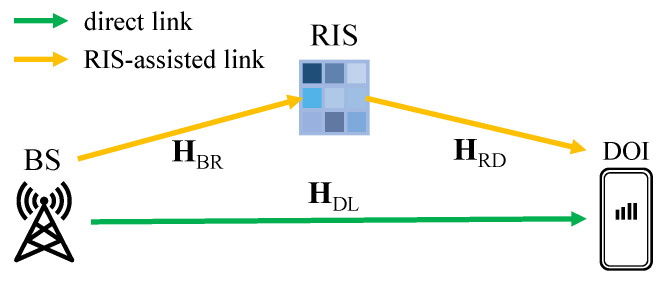
Path loss model in RIS-enhanced environments where the channel matrices H among the BS, the DOI, and the RIS are known.

The values of $G_{p}$ and $L_{p}$ are usually given in the device documentation. To calculate *PL*, we first assume that the RIS only affects the phase shifts, which means there is no loss in the amplitude of the incident signals. Next, our path loss model works in a MIMO-OFDM system with *K* sub-carriers, containing a BS with *P* antennas, a DOI with *Q* antennas, and an RIS with *M* elements. For the sub-carrier *k*, the channel matrices of the direct link $H_{DL,k}\in C^{Q\times P}$ (from the BS to the DOI) and the RIS-assisted link, including $H_{BR,k}\in C^{M\times P}$ (from the BS to the RIS) and $H_{RD,k}\in C^{Q\times M}$ (from the RIS to the DOI), are provided by the DeepMIMO dataset. Figure 5 depicts the scenario we consider for path loss calculation. According to [72], for all *K* sub-carriers, the channel matrix of the direct link $H_{DL}\in C^{Q\times P}$ is just the sum of all sub-carrier components; i.e.,

$$H_{DL}=\sum_{k=1}^{K}H_{DL,k}.$$

As defined in [73], given the codeword $\psi \in C^{M\times 1}$, the channel matrix of the RIS-assisted link $H_{RL}\in C^{Q\times P}$ over all *K* sub-carriers is calculated as:

$$H_{RL}=\sum_{k=1}^{K}H_{RD,k}diag\left(\psi \right)H_{BR,k},$$

where diag(ψ) is the diagonal matrix with the entries of ψ on its diagonal. Note that the codeword ψ is the same for all *K* sub-carriers. Finally, using the method described in [31,35], the path loss PL in decibels from the BS to the DOI in this model is

$$\mathrm{PL}\left(\mathrm{dB}\right)=10\log_{10}|H_{DL}+H_{RL}{|}^{2}=20\log_{10}\left|H_{DL}+H_{RL}\right|,$$

where |·| means the complex magnitude. Finally, combining these formulas, we can calculate the RSSI of BSs for a DOI using the DeepMIMO dataset.

Note that we do not need this RSSI calculation step in real-world deployments, since we can directly access the RSSI values using users’ devices, such as mobile phones. We emphasize that our fingerprint-based method has no dependencies on accurate field measurements. The experiments in this paper rely on simulation using the DeepMIMO because RIS hardware is currently rare. There are only a few prototypes throughout the world [66,67,68]. Hence, collecting real-world data through experiments is hardly possible at present.

### 3.2. Fingerprint-Graph Transformation

Many data-driven fingerprint-based methods utilize conventional machine learning techniques or deep learning models, which only work for Euclidean data and are ineffective for non-Euclidean data (e.g., fingerprints) [74]. In comparison, GNNs are particularly designed for non-Euclidean data and have proven to be efficient for various downstream tasks [75]. Hence, the fingerprints should be first transformed into graphs to adapt GNN-based models. In our prior work [74], we proposed a two-stage preprocessing method to achieve this.

**Figure 6:**
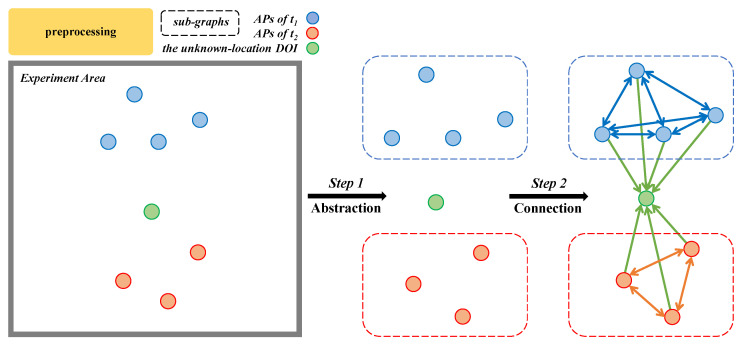
The fingerprint-graph transformation method proposed in our prior work [74], instantiated for |T|=2 as an example.

We consider an environment with |T| RF technologies, where $T=\{t_{1},t_{2},\ldots \}$ denotes the set of all RF types. Then, as illustrated in Figure 6, the transformation method is as follows.

**Step 1. Abstraction:** First, we gather information of all transmitters, including their types and locations. In the example shown in Figure 6, the transmitters are access points (APs), and $T=\{t_{1},t_{2}\}$, which means there are two types of RF signals. We consider transmitters as vertices in a graph. The green vertex is the DOI, whose location is unknown. Then, we assign the vertex features for transmitters by the combination of their locations and RSSI. Note that vertices of different types should be considered per type, which results in *heterogeneous* graphs.

Different RF technologies usually have different propagation laws, which means that encoding with the same set of model parameters is insufficient. Next, for connectivity, we first make two assumptions to decide on the adjacency between vertices.

Assumption I: Edges between vertices denote all possible signal propagations and interferences.
Assumption II: A transmitter will only affect other transmitters of the same type.

**Step 2. Connection:** Considering Assumption I, since the DOI measures RSSI from all transmitters, there must be edges between the DOI and all transmitters. Note that these edges are *unidirectional* because the DOI is only a measuring device. Assumption II implies that the RSSI from a transmitter measured by the DOI is a combined result of all transmitters of the same type. Hence, transmitters of the same type will be fully connected. Since transmitters of the same type will affect each other, the edges within each *sub*-graph are *bidirectional*.

We can also assign edge features by theoretical signal attenuation models. In [74], the log-normal shadowing model (LNSM) was adopted [76]. In this way, given an arbitrary fingerprint, this transformation method could generate a corresponding graph for GNN models. In Section 4.3, we modify this preprocessing method to generate graphs for fingerprints gathered in RIS-enhanced environments.

### 3.3. Domain Adversarial Neural Network

**Figure 7:**
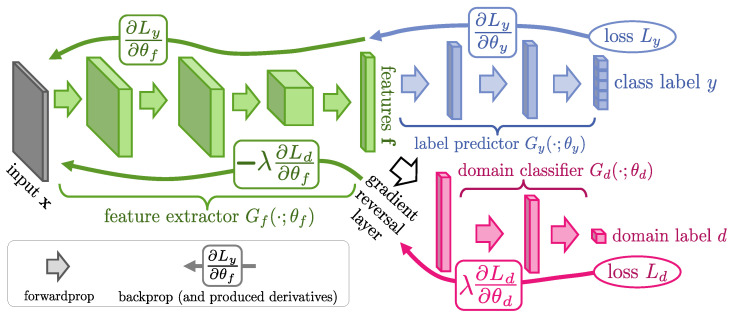
Architecture of the DANN first proposed in [37].

In Section 2.2, we discussed the DG problem and mentioned that the DANN framework fits the problem of this paper. In this section, we briefly elaborate on the theory of DANN. Figure 7 illustrates the DANN framework, containing three main parts: a feature extractor, a domain discriminator (classifier), and a label predictor, which collaborate during offline training to achieve domain generalization [37] by extracting features that are irreverent to the concerned domain. Specifically, except for minimizing the errors on the label estimation, the DANN also reverses the gradient with the help of a gradient-reversal layer (GRL) [55]. Concretely, the GRL only works during backpropagation (BP). It first obtains the gradient from the first layer of the domain discriminator, reverses the gradients from the domain discriminator by multiplying a negative number −λ(λ>0), and finally passes the reversed gradient to the subsequent layer. Consequently, the feature extractor simultaneously considers gradients from both the label predictor to minimize the estimation errors and the domain discriminator to minimize the divergence of feature distributions for different domain labels.

A well-trained feature extractor can ensure the distributions of the extracted features over different domains are as similar as possible. This way, no matter what the domain labels are, the generated data features are ideally of the same distribution.

## 4. Codeword-Independent Localization

In this section, we provide the details of our proposed localization solution, including the overall design, the offline training, the online inference pipelines, the building blocks, and their corresponding motivations.

### 4.1. Codebook Calculation

In Section 3.3, we discussed that for the DANN model’s training, except for fingerprints and corresponding locations, domain labels, i.e., the codewords $\psi^{*}$, are also required in the training dataset. Hence, we need to generate all possible RIS codewords (codebook) for experiments given $C=C_{l}^{2}$ (resolution counted in degrees) and the phase shift range *R* for RSSI calculation and domain adversarial learning. We applied the method in [73]. Suppose the RIS contains $M=M_{H}\times M_{V}$ elements. Then, for the $m_{H}$-*th* ($m_{H}\in \left[1,M_{H}\right]$) column of the discrete Fourier transform (DFT)-based codebook [77], $C_{H}\in C^{M_{H}\times C_{l}}$ for the horizontal dimension is

$$\frac{1}{\sqrt{M_{H}}}{\left[1,e^{-j\frac{m_{H}}{M_{H}}R\cdot 1},e^{-j\frac{m_{H}}{M_{H}}R\cdot 2},\ldots ,e^{-j\frac{m_{H}}{M_{H}}R\cdot \left(M_{H}-1\right)}\right]}^{T}.$$

Then, we can use it a similar way to calculate the DFT-based codebook $C_{V}\in C^{M_{V}\times C_{l}}$ for the vertical dimension. Finally, the codebook $C=\sqrt{M_{H}M_{V}}C_{H}⨂C_{V}$, where $C\in C^{M\times C}$ and ⨂ denotes the Kronecker product [78]. Each column in C is a legitimate codeword.

**Figure 8:**
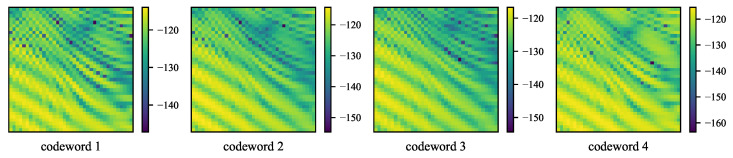
Shifted RSSI measurements (dB) using four different codewords for the same area. Each pixel denotes an RSSI value.

Codewords have a great impact on fingerprints. As shown in Figure 8, the RSSI values are different at the same locations when using different RIS codewords, which means as long as the fingerprint dataset for offline training does not contain RSSI measurements corresponding to all possible codewords, the fingerprints for online inference will have a different distribution from the training dataset. We provide our solution to this induced DG problem in this section.

### 4.2. Offline Training and Online Inference Pipelines

Before model training, our system first transforms the fingerprints into graphs, since recent studies argued that for non-Euclidean data such as fingerprint data, graph neural networks could extract more effective encodings for various downstream tasks than other models designed for Euclidean data [74,79]. The specific fingerprint-graph transformation method will be elaborated later, in Section 4.3. After we obtain the fingerprint graphs, the training process is ready.

**Figure 9:**
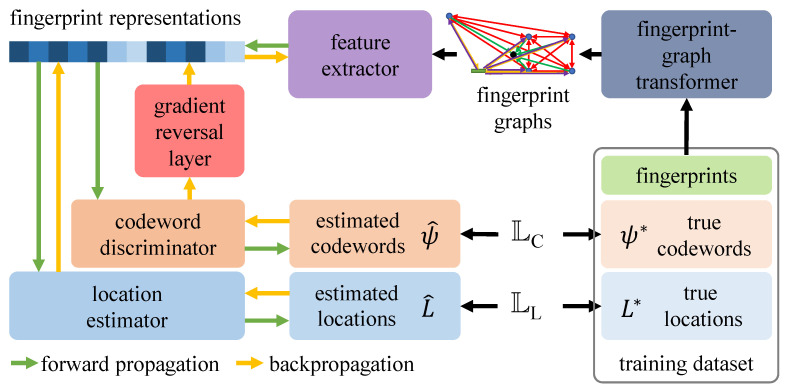
Our proposed localization system framework (for offline training).

Figure 9 illustrates the whole training pipeline. During the forward propagation, the feature extractor first encodes the fingerprint graphs and obtains their representation vectors, which will be fed to both the location estimator and the codeword discriminator for location estimation $\hat{L}$ and codeword estimation $\hat{\psi }$, respectively. To measure the errors between the estimated results and the true labels, we utilize two loss functions separately for locations and codewords. The mean squared error (MSE) [80] measures the localization error $L_{L}$ between estimated locations $\hat{L}$ and true locations $L^{*}$, i.e., $L_{L}=\mathrm{MSE}\left(\hat{L},L^{*}\right)$. Measuring the codeword errors is relatively more complex, since the codewords are complex-valued vectors, whereas conventional loss functions only support real numbers. Hence, here we adopt the complex-valued version MSE (CV-MSE) for codewords [81,82], i.e., given $\vec{c_{1}}=\vec{a_{1}}+\vec{b_{1}}i$ and $\vec{c_{2}}=\vec{a_{2}}+\vec{b_{2}}i$, where $c_{1},c_{2}\in C^{N\times 1}$,

$$\mathrm{CV}-\mathrm{MSE}\left(\vec{c_{1}},\vec{c_{2}}\right)=\frac{1}{N}\sum_{i=1}^{N}\left(\right|a_{1}\left[i\right]-a_{2}\left[i\right]{|}^{2}+|b_{1}\left[i\right]-b_{2}\left[i\right]{|}^{2}).$$

In this way, we can obtain a real-number loss to describe the estimation error for complex values. Then, for estimated codewords $\hat{\psi }$ and true codewords $\psi^{*}$, the codeword estimation error is as follows: $L_{C}=\mathrm{CV}-\mathrm{MSE}\left(\hat{\psi },\psi^{*}\right)$. The whole forward propagation process is denoted by green arrows in Figure 9. The backpropagation (BP) process was already discussed in Section 3.3 and is denoted by yellow arrows in Figure 9.

There are some additional concerns about the negative constant −λ(λ>0) we mentioned in Section 3.3. During the initial phase of training, the feature extractor is not well-trained, so at this stage, the reversed gradient from the GRL should be suppressed, i.e., λ closing in on zero. Then, as the training proceeds, the feature extractor could gradually extract meaningful representation vectors; thus, the importance of adversarial learning on the domain is growing. Therefore, λ should gradually grow from zero. Supposing p∈[0,1] denotes the training progress, then instead of a fixed λ, we can define a more flexible version $\lambda_{p}$ [55]:

$$\lambda_{p}=\frac{2}{1+exp(-\gamma *p)}-1,$$

where we set γ=10 by default in the following experiments. $\lambda_{p}$ would gradually grow from 0 to 1 as the training proceeds.

**Figure 10:**
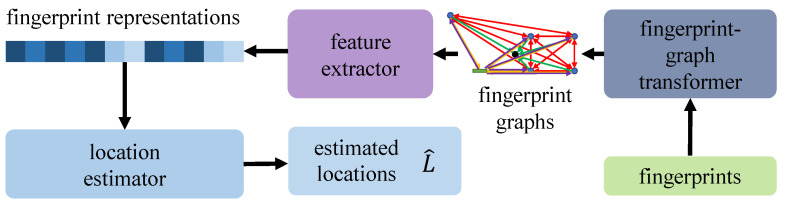
The online inference pipeline of our proposed system.

The feature extractor is theoretically capable of obtaining codeword-independent representations after a proper training process. Hence, thanks to the adversarial learning on the RIS codeword domain by the DANN framework, during online inference, the codewords are unnecessary for location estimation, which meets our requirement theoretically. Figure 10 depicts the online inference pipeline of the proposed system. Compared with the training pipeline, the codeword discriminator branch is non-essential, so it was removed.

### 4.3. Fingerprint-Graph Transformer

We extend the fingerprint-graph transformer of our prior work [74] described in Section 3.2 [74] by considering the RIS as vertices in graphs. We first define the localization scenario. Suppose a communication system operates in frequencies $F=\{f_{1},f_{2},\ldots \}$. For each f∈F, there are several BSs $B_{f}=\left\{b_{f}^{1},b_{f}^{2},\ldots \right\}$ whose locations L(·) are known. The DOI can measure the RSSI for all BSs operating in all frequencies. Finally, there is an RIS in this system whose function is unknown, whereas we know its location. Now we can start to build graphs.

**Figure 11:**
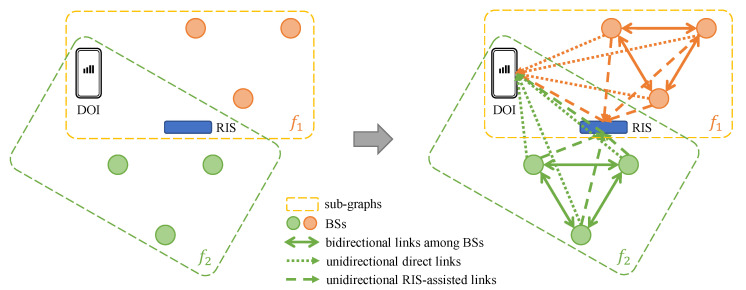
A fingerprint-graph transformation example for $F=\{f_{1},f_{2}\}$.

We consider the BSs, the RISs, and the DOIs as vertices in a graph. Then, we can construct a sub-graph for every f∈F, as shown in the left part of Figure 11, including $B_{f}$, the RIS, and the DOI. Note that the DOI and the RIS are shared among sub-graphs. The BSs in different *f* should be categorized as different kinds of vertices, inducing heterogeneous graphs when |F|>1. Actually, the RF signals of type *T* in Section 3.2 and the operating frequency *F* here play the same role—i.e., as a means of discrimination between different sub-graphs. This is because, for different *f*, the signal propagation laws are still different, which also require different sets of model parameters to encode. The vertex features for every BS *b* are $\left[RSSI_{b},L\left(b\right)\right]$, where $RSSI_{b}$ is the RSSI value of *b* measured by the DOI. We can also assign vertex features of the RIS by L(RIS).

Next, we consider the connectivity between vertices. The right part of Figure 11 illustrates the edges among all vertices. Following the assumptions of Section 3.2, we fully connect $B_{f}$ within each sub-graph by bidirectional edges. For the direct links, the edges are unidirectional from BSs to the DOI, as the DOI is a measuring device here. Similarly, the RIS is also a (nearly) passive device [83], so the RIS-assisted links are also unidirectional from the BSs to the RIS and from the RIS to the DOI. In doing so, these edges represent all possible signal propagations and interferences in this communication system. Additionally, for the edges among BSs and edges from BSs to the RIS, we also assign edge features by log-normal shadowing model (LNSM) (see Section 3.2), indicating the theoretical relative strength of signal attenuation in between.

This way, given arbitrary fingerprints, we can apply the transformation method to generate a corresponding heterogeneous graph containing |F| sub-graphs, which is ready to be fed into the GNN-based feature extractor. Some may argue that this preprocessing method involves locations of BSs and DOI, which results in additional communication burdens compared with transmitting RIS codewords. However, we can easily represent the locations by 3-axis coordinates, which are much simpler than large CV vectors of codewords. Meanwhile, the locations of BSs and RIS are unlikely to change very often; thus, infrequent updates are sufficient.

### 4.4. Feature Extractor

**Figure 12:**
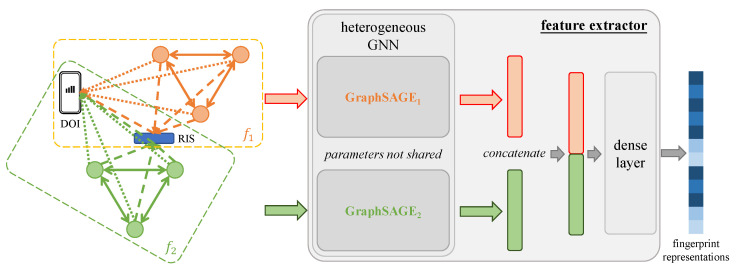
The feature extractor for the fingerprint graphs from Figure 11.

The fingerprint graphs generated by the transformer are heterogeneous, and thereby we need a heterogeneous GNN-based model to encode them. Specifically, as shown in Figure 12, for each sub-graph, we first respectively assign a GraphSAGE [84] model using pooling aggregators by default to obtain the corresponding sub-graph-wise readout by the mean of latent features of all vertices. The reason for choosing the GraphSAGE to encode each sub-graph is we can manually set the aggregation depth for neural message passing [85] and randomly select several paths among all possible ones to reduce the complexity due to potential numerous vertices (BSs) and edges (communication links) in our fingerprint graphs [84]. Then, all sub-graph-wise readouts are concatenated to a vector, which will be fed into a dense layer for the fingerprint representations. We utilize leaky ReLU [86] as the activation functions for the feature extractors, for which the negative slope coefficient was −0.02 for all the following experiments.

### 4.5. Location Estimator

The fingerprint-graph transformer and the feature extractor cooperate to generate fingerprint representations. To further obtain the location estimation, we need to apply the location estimator to read out the fingerprint representations. In our system, we simply adopt a three-layer perceptron as the location estimator activated by the leaky ReLU [86].

### 4.6. Codeword Discriminator

As the codewords $\psi^{*}$ are CV vectors, the codeword discriminator should support CV outputs. Hence, we implement dense layers that accept both real-value (RV) and CV inputs and output CV vectors with the help of the *cplxmodule* library [87]. This way, we can build a multi-layer perceptron (MLP) of CVs for the codeword discriminator. Additionally, the activation function should also support these CV-adapted layers. Therefore, we utilize *modReLU* [88], i.e., a variant of ReLU designed for pointwise nonlinearity that only manipulates the magnitudes of the CV inputs, to activate the codeword discriminator in our system. For c∈C,

$$modReLU\left(c\right)=ReLU\left(|c|+b\right)\times \frac{c}{\left|c\right|},$$

where b∈R is a bias parameter of the nonlinearity. In other words, *b* is a threshold to decide whether to make the activated *c* equal to zero. We set b=0.5 by default.

As mentioned before, the codewords describe the phase shifts of all RIS elements. As such, instead of using complex numbers to represent the codewords, we can use the RV phase shifts of all RIS elements by radians. Then, additional modifications for CV supports are unnecessary for the codeword discriminator. However, complex numbers are widely used in signal processing and electrical engineering, as they provide convenient representations for the phases and amplitudes of periodic signals [89,90]. Real numbers are not straightforward to use to represent this information. Hence, using real numbers to represent codewords would make it difficult for the codeword discriminator to learn from the data and provide reliable adversarial gradients for the DANN framework [91,92]. Our experiments in Section 5 demonstrate that the CV version of our proposed solution performs better than the RV version.

## 5. Evaluation

In this section, we first introduce the settings of the DeepMIMO for our evaluation. Next, the performances of our proposed system are given, including the experiments on the impacts of crucial system’s parameters to demonstrate the robustness of our method.

### 5.1. Experimental Setup

**Figure 13:**
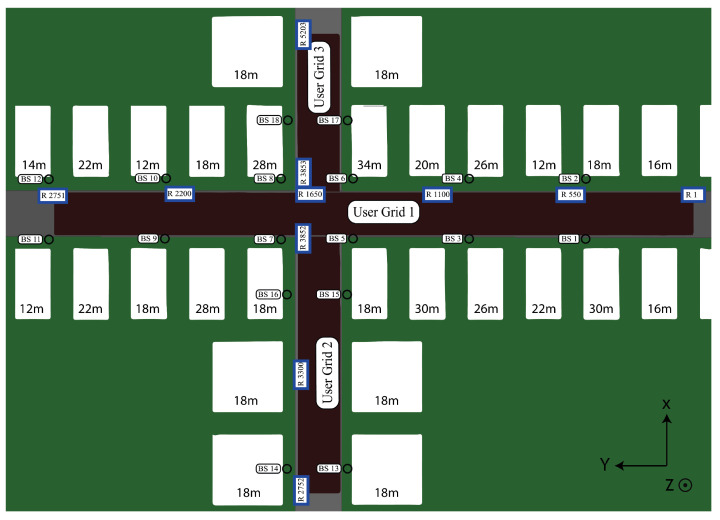
The O1 scenario in the DeepMIMO [38].

For our experiments, we used the dataset generated by the DeepMIMO [38]. The dataset is a generative dataset based on ray-tracing measurements, which is semi-customizable by user specifications of the system’s parameters [38]. Our experiments were fully conducted considering the O1 (Outdoor 1) scenario of the DeepMIMO, as shown in Figure 13. Concretely, we are interested in a modern wireless communication network containing both 4G and 5G. Hence, among all available operating frequency choices in the DeepMIMO, we considered 3.4 GHz, 3.5 GHz (4G LTE Band 42 [93]), and 28 GHz (5G NR FR2 Band n257 [94]). We activated six BSs in the DeepMIMO simulation tool, where the BS5 plays the role of RIS using the same method discussed in [73]. We specified the BSs, RIS, and DOI, all equipped with MIMO antennas. Detailed settings are listed in Table 1.

**Figure 14:**
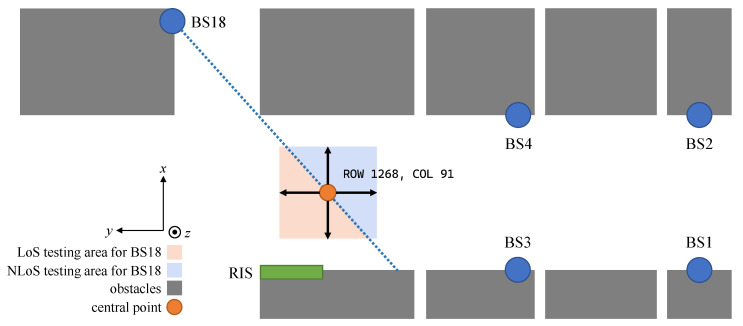
A top-view sketch map of the experimental area using the O1 scenario in the DeepMIMO.

Considering the positions of BSs and obstacles, we specified the test areas of DOI containing both LoS and NLoS regions for BS18, as shown in Figure 14. Taking the testing point (ROW 1268, COL 91) as the center, we could equidistantly expand the boundaries to obtain test areas, where half is LoS and the other half is NLoS for BS18. We tested the localization performances in the testing area.

### 5.2. Experimental Parameters

We considered the number of codewords *C* and the size of test areas *A* and tested the robustness of our localization system by manually adding extra additive white Gaussian noise (AWGN) $N∼N(0,\sigma^{2})$ to the calculated RSSI, where N means Gaussian distribution and σ is the variance [95]. As such, we can use a parameter set {C,A,σ} to describe the experiments. The AWGN here refers to some miscellaneous noises, including device noise [96], fading [97], and polarization mismatch [98], which are not discussed in Section 3.1.1 and are able to be modeled by Gaussian processes [99,100].

In Section 3.1, we mentioned that most RIS prototypes only support C=2/4 [66,67], whereas others may realize C=64 [68]. For forward-looking results in this paper, we set an initial resolution of 15°, and R= 180°. Then, we had C=144. We set A=51.84 and σ=0 by default. We first applied the default experimental parameter set {C=144,A=51.84,σ=0} for evaluation to check the feasibility of our solution. Then, we changed one parameter and kept the other two unchanged to separately investigate the impacts of these three experimental parameters on the proposed solution.

### 5.3. Dataset Generation and Model Implementation

Given the experimental parameters {C,A,σ}, we first applied the formulas in Section 4.1 to generate the codebook C using *C*. Next, we input the settings in Table 1 and *A* to the DeepMIMO dataset generator by modifying the parameters.m file, which is one of the supporting scripts provided by the DeepMIMO [38]. Then, after running another script DeepMIMO_Dataset_Generator.m, we obtained the channel matrices among BSs, DOI, and the RISs, along with the corresponding DOI locations, which are the labels in our problem. Finally, by applying the RSSI calculation pipeline presented in Section 3.1.1 using C and σ, we calculated the RSSI values, thereby constructing fingerprint datasets for evaluation. Here, we can simply assume that for all BSs we activated, $G_{p}=40$ dB and $L_{p}=20$ dB. We implemented these operations using MATLAB R2022a.

For the training/test split, we first randomly sampled 80% of available codewords, then randomly sampled 80% locations in the testing area. Then, a data point, whose location and codeword were sampled, belonged to the training dataset. Conversely, if neither its location nor codeword was sampled, it was assigned to the test datasets. In other words, we tested the estimation model using fingerprints of unknown locations and unknown codewords. This way, the test data for online inference challenged the generalization capabilities for both locations and codewords.

We implemented the proposed system using PyTorch [101] with the help of the DGL library [102] to build graph models for the feature extractor. All neural network layers contained 64-dimensional latent features, resulting in a training model with around 370,000 trainable parameters (float32). The whole system was trained using an Adam optimizer [103] with an initial learning rate of 0.01. We trained models and obtained simulation results on a laptop built by Dell Inc., Round Rock, TX, USA with an Intel(R) Core(TM) i7-10750H CPU and an NVIDIA GeForce GTX 1650 Ti graphic card. Using this hardware set, we easily achieved approx. 820 online inference per second without dedicated optimization.

### 5.4. Oracle and Baseline Cases for Evaluation

The goal of this paper is to solve the codeword domain generalization problem. The best possible model completely eliminates the negative effects of the DG issue. Hence, we simulated this case by manually setting C=1, which means there was only one possible RIS codeword. Then, there were no fingerprint shifts because the codeword domains for both training and testing were always identical. We call this case the *oracle case*, as this is the theoretical upper bound of our localization system.

On the other hand, the worst possible model completely fails to decouple the correlation between fingerprints and corresponding codewords, which is equivalent to applying no adversarial learning. In this case, for the same location, there will be multiple different fingerprints corresponding to it due to different RIS codewords. The localization system will have difficulty learning effective fingerprint representations from such data. We call this case the *baseline case*, since this is the theoretical lower bound of the proposed system. If the performance of our solution better resembles the *oracle case* rather than the *baseline case*, then we can conclude that our model has successfully learned codeword-independent representations of fingerprints.

We implemented these two cases in our solution simply by removing the codeword discriminator component because neither of them requires adversarial learning on the codewords. In the following experiments, all parameters {C,A,σ} in the *baseline case* remained the same as in the test scenario. For the *oracle case*, *C* always equals 1, so we kept only *A* and σ the same.

### 5.5. Performance Evaluation

**Figure 15:**
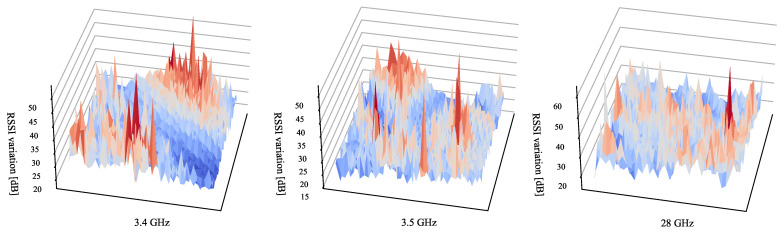
Fingerprint shifts (dB) in the testing area for three frequencies. The height means the maximum RSSI shift at that position when {C=144,A=51.84,σ=0}.

We applied the default experimental parameter set {C=144,A=51.84,σ=0} for evaluation to check the feasibility of our solutions. Figure 15 illustrates fingerprint shifts in the testing area. RSSI values at the same location can differ by up to 52.1 dB, which indicates that the fingerprints are sensitive to codeword change, thereby demonstrating the feasibility of using the DeepMIMO for our evaluation.

To visualize the location-estimation errors, we used bar charts: each bar’s middle line is the mean squared error (MSE), and its height equals two times the standard deviation (Var) over the mean squared error of all concerned testing points. Most errors are within this interval, which indicates the prediction stability of each method. The numerical results are also provided in tables.

**Figure 16:**
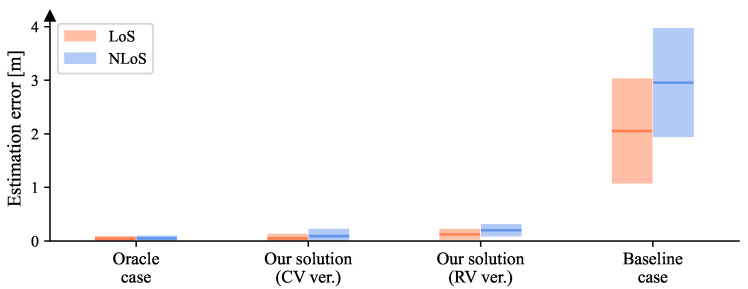
The location-estimation errors of both LoS and NLoS areas for the oracle/baseline cases, and the CV/RV versions of our solution when {C=144,A=51.84,σ=0}. Given the squared error set *e*, for each bar, the middle line is mean(e), i.e., MSE, and its height ranges from MSE - std(e) to MSE + std(e).

For the experimental parameter set {C=144,A=51.84,σ=0}, the results of the oracle/baseline cases and our solution are shown in Figure 16. One may notice that the location-estimation errors of our solution are much closer to that of the oracle case rather than the baseline case, which illustrates the representations extracted by our method accommodate different codewords. Therefore, we can conclude that the feature extractor in our system can obtain codeword-independent representations of fingerprints. Then, compared with the RV version, the desired CV version of our method obtained smaller errors, which supports our analysis in Section 4.6. The detailed numerical results presented in Table 2 show that we achieved centimeter-level accuracy.

### 5.6. Impacts of Experimental Parameters

In this section, we investigate the impact of experiment parameters on the model’s performance by changing one parameter and keeping another two fixed. To visualize the location-estimation errors, we use bar charts: each bar’s middle line is the mean squared loss (MSE) [80], and its height equals two times the standard deviation over the squared loss of all concerned testing points. Most of the errors are within this interval, which could be used to observe the prediction stability of each method. Corresponding numerical results (both MSE and variance) are provided in tables.

#### 5.6.1. Impact of Number of Codewords

**Figure 17:**
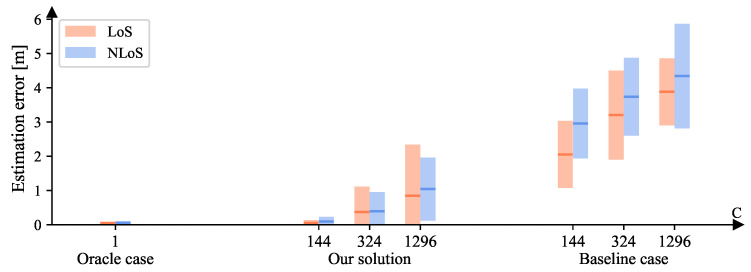
The location-estimation errors of both LoS and NLoS areas for the oracle/baseline cases and our solution when {C=144/324/1296,A=51.84,σ=0}. Given the squared error set *e*, for each bar, the middle line is mean(e), i.e., MSE, and its height ranges from MSE −std(e) to MSE + std(e). Note that for the oracle case, *C* always equals 1 for reference.

The resolution of the RIS in previous experiments was set to $15^{\circ }$, inducing C=144, which is already far more than what current RIS prototypes can provide. Here, we further enlarged *C* for more harsh scenarios by assuming smaller resolutions, i.e., $10^{\circ }$ and $5^{\circ }$, obtaining C=324 and C=1296, respectively. Note that for the oracle case, *C* always equals one for the purpose of benchmarking. Other parameters remained unchanged in this section—i.e., A=51.84 and σ=0. Figure 17 illustrates the location-estimation errors of the oracle/baseline cases and our solution. As *C* increases, both the MSE and the standard deviations become larger, as the feature extractor has to adapt to more codewords at the same time. Nonetheless, the performance of our solution is still nearer to that of the oracle case than the baseline case. When C=1296, our solution even performs better than the baseline case when C=144. These performances demonstrate that our solution maintains its capability of being codeword-independent when *C* becomes much larger than current prototypes. This proves the robustness to the number of available codewords. Table 3 presents the corresponding numerical results.

#### 5.6.2. Impact of Testing Area Size

The testing area size *A* in previous experiments was always 51.84 m${}^{2}$. Instead, we set it to A=51.84/92.16/144.00 and investigated its impact on the system’s performance. We kept C=144 and σ=0.

**Figure 18:**
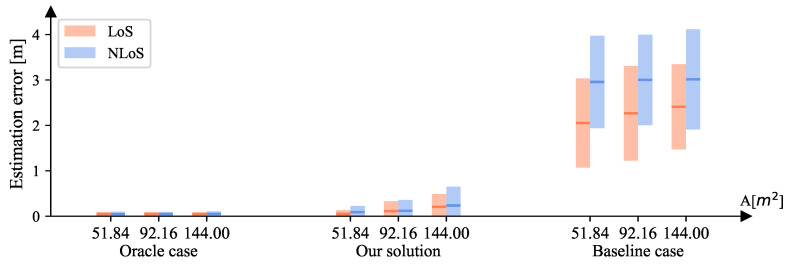
The location-estimation errors of both LoS and NLoS areas for the oracle/baseline cases and our solution when {C=144,A=51.84/92.16/144.00,σ=0}. Given the squared error set *e*, for each bar, the middle line is mean(e), i.e., MSE, and its height ranges from MSE −std(e) to MSE + std(e).

Figure 18 depicts the performances of the oracle/baseline cases and our solution. The performances of the oracle and baseline cases almost remained the same as *A* increased, whereas the location-estimation error of our solution grew faster than the reference cases. However, this phenomenon is reasonable because our solution needs to perform adversarial learning on more testing points. This means that it becomes harder for the feature extractor to obtain codeword-independent representations as the number of fingerprints grows. Both the oracle and baseline cases perform no adversarial learning; hence, their performance remained nearly unchanged. Nevertheless, our solution still achieved significantly better results than the baseline case and is closer to the oracle case. Therefore, we conclude that our solution is robust against changes in the testing area size. All corresponding numerical results are presented in Table 4.

#### 5.6.3. Impact of Additive White Gaussian Noise (AWGN)

We manually added extra AWGN to the calculated RSSI to validate the stability of our solution. The value of the AWGN N (dB) was controlled by the standard deviation σ for $N∼N(0,\sigma^{2})$. One may note that the extra AWGN here was not caused by obstacles in the environments, which were already recorded by the channel matrices in the DeepMIMO dataset and implicitly represented by the calculated RSSI. We investigated AWGN here for simulation of device noise to further refine our results. Numerical results are presented in Table 5.

**Figure 19:**
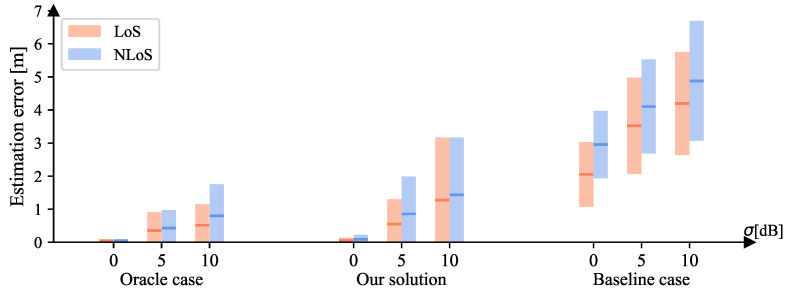
The location-estimation errors of both LoS and NLoS areas for the oracle/baseline cases and our solution when {C=144,A=51.84,σ=0/5/10}. Given the squared error set *e*, for each bar, the middle line is mean(e), i.e., MSE, and its height ranges from MSE −std(e) to MSE + std(e).

We investigated the performances when σ=0/5/10. We kept C=144 and A=51.84. Figure 19 shows the location-estimation errors of the oracle/baseline cases and our solution for different AWGN values. It can be seen that our solution performs much better than the baseline cases and is more similar to the oracle cases.

## 6. Conclusions and Future Work

In this paper, we first investigated the localization problem in modern RIS-enhanced wireless communication networks. Model-driven methods usually rely on sophisticated communication models and set specific optimization goals to realize accurate localization. However, the complexity of these models grows fast as the numbers of BSs and DOI increase. This restricts their application scope. Although several data-driven methods have addressed this problem, they unrealistically assumed that the RIS is solely deployed for localization. Both model- and data-driven methods share the same drawbacks in that they require the RIS codewords for location inference, which induces a huge additional communication burden.

These observations inspired us to design a localization solution for RIS-enhanced environments which can decouple the correlation between fingerprints and codewords and be code-word independent. We designed a pre-processing step to transform the fingerprints into graphs for the heterogeneous GNN-based feature extractor to make full use of the non-Euclidean features of the fingerprints, especially when they are collected in environments with multiple operating frequencies. Our offline training pipeline enabled the feature extractor to generate the representations of the fingerprint graphs and feed them into both the MLP-based location estimator and the CV-MLP codeword discriminator for location and codeword estimation, respectively. Then, the gradients from the codeword discriminator were reversed by a gradient reversal layer to perform adversarial learning on the codeword domain during backpropagation to ensure the distributions of the representations for different codewords are as similar as possible. By doing so, the feature extractor could extract codeword-independent features for location inference, thereby eliminating the need to have the codewords for the online inference stage.

We evaluated our system using the O1 scenario in the DeepMIMO dataset. We defined oracle and baseline cases for a fair comparison with our solution and elaborated the method to calculate the RSSI using the channel matrices given in the DeepMIMO simulation tool. Our evaluation results showed that our localization is (i) codeword-independent and (ii) robust against changes in the number of codewords, testing area size, and additive white Gaussian noise. The experiments also showed that complex-value codeword discriminators performed better than real-value codeword discriminators.

Although we used a far larger number of the codewords compared to the current RIS prototypes [66,67,68] and achieved performances close to those of the oracle cases, there is still a chance that our model fails to deal with more codewords when the resolution is extremely low. If it happens, then we may have to require the codewords for location inference. Nevertheless, considering the fact that most currently available prototypes only support C=2/4 [66,67], we are confident that our solution has strong practicality in modern wireless communication systems.

For our evaluation, we fully relied on simulations using the DeepMIMO dataset, as the RIS hardware is currently still under development, even though some prototypes exist. Hence, in our future works, if RIS hardware becomes available, we will design experiments, collect real-world data, and perform the evaluation.

## Figures and Tables

**Table 1:** DeepMIMO settings for our experiments.

Operating frequency	3.4 GHz, 3.5 GHz, 28 GHz
Activated BSs	1, 2, 3, 4, 5 (RIS), 18
Antennas of BSs and RIS	4 × 4
Antennas of DOI	2 × 2
Bandwidth	200 MHz
The number of OFDM sub-carriers	512

**Table 2:** The localization errors (*m*) of LoS (orange) and NLoS (blue) areas for the oracle/baseline cases, and the CV/RV versions of our solution when {C=144,A=51.84,σ=0}.

	LoS		NLoS	
	MSE	Var	MSE	Var
Oracle case	0.045	0.002	0.047	0.003
Our solution (CV ver.)	0.050	0.007	0.090	0.018
Our solution (RV ver.)	0.125	0.010	0.199	0.013
Baseline case	2.053	0.958	2.956	1.033

**Table 3:** The localization errors (*m*) of LoS (orange) and NLoS (blue) areas for the oracle/baseline cases and our solution when {C=144/324/1296,A=51.84,σ=0}. Note that for the oracle case, *C* always equals to 1 for reference.

Oracle Case (C=1)					Our Solution				Baseline Case			
**LoS**		**NLoS**		C	**LoS**		**NLoS**		**LoS**		**NLoS**	
**MSE**	**Var**	**MSE**	**Var**		**MSE**	**Var**	**MSE**	**Var**	**MSE**	**Var**	**MSE**	**Var**
0.045	0.002	0.047	0.003	144	0.050	0.007	0.090	0.018	2.053	0.958	2.956	1.033
				324	0.368	0.554	0.394	0.312	3.202	1.692	3.737	1.296
				1296	0.843	2.231	1.039	0.845	3.882	0.958	4.342	2.333

**Table 4:** The localization errors (*m*) of LoS (orange) and NLoS (blue) areas for the oracle/baseline cases and our solution when {C=144,A=51.84/92.16/144.00,σ=0}.

	Oracle Case				Our Solution				Baseline Case			
A **[m^2^]**	**LoS**		**NLoS**		**LoS**		**NLoS**		**LoS**		**NLoS**	
	**MSE**	**Var**	**MSE**	**Var**	**MSE**	**Var**	**MSE**	**Var**	**MSE**	**Var**	**MSE**	**Var**
51.84	0.045	0.002	0.047	0.003	0.050	0.007	0.090	0.018	2.053	0.958	2.956	1.033
92.16	0.045	0.002	0.047	0.002	0.113	0.048	0.119	0.056	2.268	1.086	3.002	0.991
144.00	0.046	0.002	0.050	0.003	0.205	0.081	0.236	0.170	2.410	0.879	3.013	1.211

**Table 5:** The localization errors (*m*) of LoS (orange) and NLoS (blue) areas for the oracle/baseline cases and our solution when {C=144,A=51.84,σ=0/5/10}.

	Oracle Case				Our Solution				Baseline Case			
σ **[dB]**	**LoS**		**NLoS**		**LoS**		**NLoS**		**LoS**		**NLoS**	
	**MSE**	**Var**	**MSE**	**Var**	**MSE**	**Var**	**MSE**	**Var**	**MSE**	**Var**	**MSE**	Var
0	0.045	0.002	0.047	0.003	0.050	0.007	0.090	0.018	2.053	0.958	2.956	1.033
5	0.353	0.315	0.430	0.298	0.552	0.556	0.858	1.280	3.522	2.118	4.104	2.022
10	0.517	0.402	0.802	0.910	1.279	3.569	1.435	2.994	4.200	2.430	4.881	3.291

## Data Availability

Data were generated using the open-source DeepMIMO [38].

## References

[1] Welkie A., Shangguan L., Gummeson J., Hu W., Jamieson K. Programmable radio environments for smart spaces. Proceedings of the 16th ACM Workshop on Hot Topics in Networks.

[2] Liang Y.C., Chen J., Long R., He Z.Q., Lin X., Huang C., Liu S., Shen X.S., Di Renzo M. (2021). Reconfigurable intelligent surfaces for smart wireless environments: Channel estimation, system design and applications in 6G networks. Sci. China Inf. Sci..

[3] Basar E. (2020). Reconfigurable intelligent surface-based index modulation: A new beyond MIMO paradigm for 6G. IEEE Trans. Commun..

[4] Lin Z., Niu H., An K., Wang Y., Zheng G., Chatzinotas S., Hu Y. (2022). Refracting RIS aided hybrid satellite-terrestrial relay networks: Joint beamforming design and optimization. IEEE Trans. Aerosp. Electron. Syst..

[5] Zhu B.O., Zhao J., Feng Y. (2013). Active impedance metasurface with full 360 reflection phase tuning. Sci. Rep..

[6] Björnson E., Wymeersch H., Matthiesen B., Popovski P., Sanguinetti L., de Carvalho E. (2022). Reconfigurable intelligent surfaces: A signal processing perspective with wireless applications. IEEE Signal Process. Mag..

[7] Huang C., Hu S., Alexandropoulos G.C., Zappone A., Yuen C., Zhang R., Di Renzo M., Debbah M. (2020). Holographic MIMO surfaces for 6G wireless networks: Opportunities, challenges, and trends. IEEE Wirel. Commun..

[8] Liu Y., Liu X., Mu X., Hou T., Xu J., Di Renzo M., Al-Dhahir N. (2021). Reconfigurable intelligent surfaces: Principles and opportunities. IEEE Commun. Surv. Tutor..

[9] Renzo M.D., Debbah M., Phan-Huy D.T., Zappone A., Alouini M.S., Yuen C., Sciancalepore V., Alexandropoulos G.C., Hoydis J., Gacanin H. (2019). Smart radio environments empowered by reconfigurable AI meta-surfaces: An idea whose time has come. EURASIP J. Wirel. Commun. Netw..

[10] Elayan H., Amin O., Shubair R.M., Alouini M.S. Terahertz communication: The opportunities of wireless technology beyond 5G. Proceedings of the 2018 International Conference on Advanced Communication Technologies and Networking (CommNet).

[11] Chowdhury M.Z., Shahjalal M., Ahmed S., Jang Y.M. (2020). 6G wireless communication systems: Applications, requirements, technologies, challenges, and research directions. IEEE Open J. Commun. Soc..

[12] Hillger P., van Delden M., Thanthrige U.S.M., Ahmed A.M., Wittemeier J., Arzi K., Andree M., Sievert B., Prost W., Rennings A. (2020). Toward mobile integrated electronic systems at THz frequencies. J. Infrared Millim. Terahertz Waves.

[13] Uwaechia A.N., Mahyuddin N.M. (2020). A comprehensive survey on millimeter wave communications for fifth-generation wireless networks: Feasibility and challenges. IEEE Access.

[14] Alkhateeb A., El Ayach O., Leus G., Heath R.W. (2014). Channel estimation and hybrid precoding for millimeter wave cellular systems. IEEE J. Sel. Top. Signal Process..

[15] Taha A., Alrabeiah M., Alkhateeb A. Deep learning for large intelligent surfaces in millimeter wave and massive MIMO systems. Proceedings of the 2019 IEEE Global communications conference (GLOBECOM).

[16] He J., Wymeersch H., Sanguanpuak T., Silvén O., Juntti M. Adaptive beamforming design for mmWave RIS-aided joint localization and communication. Proceedings of the 2020 IEEE Wireless Communications and Networking Conference Workshops (WCNCW).

[17] Karasik R., Simeone O., Di Renzo M., Shitz S.S. Beyond max-SNR: Joint encoding for reconfigurable intelligent surfaces. Proceedings of the 2020 IEEE International Symposium on Information Theory (ISIT).

[18] Dargie W., Poellabauer C. (2010). Fundamentals of Wireless Sensor Networks: Theory and Practice.

[19] Yang Z., Zhou Z., Liu Y. (2013). From RSSI to CSI: Indoor localization via channel response. ACM Comput. Surv. (CSUR).

[20] Zafari F., Gkelias A., Leung K.K. (2019). A survey of indoor localization systems and technologies. IEEE Commun. Surv. Tutor..

[21] Elzanaty A., Guerra A., Guidi F., Alouini M.S. (2021). Reconfigurable intelligent surfaces for localization: Position and orientation error bounds. IEEE Trans. Signal Process..

[22] Dardari D., Decarli N., Guerra A., Guidi F. (2021). LOS/NLOS near-field localization with a large reconfigurable intelligent surface. IEEE Trans. Wirel. Commun..

[23] Raleigh G.G., Cioffi J.M. (1998). Spatio-temporal coding for wireless communication. IEEE Trans. Commun..

[24] Paulraj A.J., Gore D.A., Nabar R.U., Bolcskei H. (2004). An overview of MIMO communications-a key to gigabit wireless. Proc. IEEE.

[25] Stuber G.L., Barry J.R., Mclaughlin S.W., Li Y., Ingram M.A., Pratt T.G. (2004). Broadband MIMO-OFDM wireless communications. Proc. IEEE.

[26] Ng D.W.K., Lo E.S., Schober R. (2012). Energy-efficient resource allocation in OFDMA systems with large numbers of base station antennas. IEEE Trans. Wirel. Commun..

[27] Wu C., Yang Z., Liu Y., Xi W. (2012). WILL: Wireless indoor localization without site survey. IEEE Trans. Parallel Distrib. Syst..

[28] Ibrahim M., Torki M., ElNainay M. CNN based indoor localization using RSS time-series. Proceedings of the 2018 IEEE symposium on computers and communications (ISCC).

[29] Abbas M., Elhamshary M., Rizk H., Torki M., Youssef M. WiDeep: WiFi-based accurate and robust indoor localization system using deep learning. Proceedings of the 2019 IEEE International Conference on Pervasive Computing and Communications (PerCom).

[30] Chen Z., Zou H., Yang J., Jiang H., Xie L. (2019). WiFi fingerprinting indoor localization using local feature-based deep LSTM. IEEE Syst. J..

[31] Zhang H., Hu J., Zhang H., Di B., Bian K., Han Z., Song L. (2020). Metaradar: Indoor localization by reconfigurable metamaterials. IEEE Trans. Mob. Comput..

[32] Pan C., Ren H., Wang K., Kolb J.F., Elkashlan M., Chen M., Di Renzo M., Hao Y., Wang J., Swindlehurst A.L. (2021). Reconfigurable intelligent surfaces for 6G systems: Principles, applications, and research directions. IEEE Commun. Mag..

[33] Huang S., Wang B., Zhao Y., Luan M. (2022). Near-Field RSS-Based Localization Algorithms Using Reconfigurable Intelligent Surface. IEEE Sens. J..

[34] Sauter M. (2010). From GSM to LTE: An Introduction to Mobile Networks and Mobile Broadband.

[35] Zhang H., Zhang H., Di B., Bian K., Han Z., Song L. (2020). Towards ubiquitous positioning by leveraging reconfigurable intelligent surface. IEEE Commun. Lett..

[36] Zhou K., Liu Z., Qiao Y., Xiang T., Loy C.C. (2022). Domain generalization: A survey. IEEE Trans. Pattern Anal. Mach. Intell..

[37] Ganin Y., Ustinova E., Ajakan H., Germain P., Larochelle H., Laviolette F., Marchand M., Lempitsky V. (2016). Domain-adversarial training of neural networks. J. Mach. Learn. Res..

[38] Alkhateeb A. DeepMIMO: A Generic Deep Learning Dataset for Millimeter Wave and Massive MIMO Applications. Proceedings of the Information Theory and Applications Workshop (ITA).

[39] Wymeersch H., Denis B. Beyond 5G wireless localization with reconfigurable intelligent surfaces. Proceedings of the ICC 2020-2020 IEEE International Conference on Communications (ICC).

[40] Rissanen J.J. (1996). Fisher information and stochastic complexity. IEEE Trans. Inf. Theory.

[41] He J., Wymeersch H., Kong L., Silvén O., Juntti M. Large intelligent surface for positioning in millimeter wave MIMO systems. Proceedings of the 2020 IEEE 91st Vehicular Technology Conference (VTC2020-Spring).

[42] Smith S. (2005). Covariance, subspace, and intrinsic Crame/spl acute/r-Rao bounds. IEEE Trans. Signal Process..

[43] Yang Z., Dai Z., Yang Y., Carbonell J., Salakhutdinov R.R., Le Q.V. Xlnet: Generalized autoregressive pretraining for language understanding. Proceedings of the 33rd International Conference on Neural Information Processing Systems.

[44] Caruana R. (1997). Multitask learning. Mach. Learn..

[45] Pan S.J., Yang Q. (2009). A survey on transfer learning. IEEE Trans. Knowl. Data Eng..

[46] Weiss K., Khoshgoftaar T.M., Wang D. (2016). A survey of transfer learning. J. Big Data.

[47] Wang J., Lan C., Liu C., Ouyang Y., Qin T., Lu W., Chen Y., Zeng W., Yu P. (2022). Generalizing to unseen domains: A survey on domain generalization. IEEE Trans. Knowl. Data Eng..

[48] Vilalta R., Drissi Y. (2002). A perspective view and survey of meta-learning. Artif. Intell. Rev..

[49] Hospedales T., Antoniou A., Micaelli P., Storkey A. (2021). Meta-learning in neural networks: A survey. IEEE Trans. Pattern Anal. Mach. Intell..

[50] Finn C.B. (2018). Learning to Learn with Gradients. Ph.D. Thesis.

[51] Huisman M., Van Rijn J.N., Plaat A. (2021). A survey of deep meta-learning. Artif. Intell. Rev..

[52] Shorten C., Khoshgoftaar T.M. (2019). A survey on image data augmentation for deep learning. J. Big Data.

[53] Bengio Y., Courville A., Vincent P. (2013). Representation learning: A review and new perspectives. IEEE Trans. Pattern Anal. Mach. Intell..

[54] Muandet K., Balduzzi D., Schölkopf B. Domain generalization via invariant feature representation. Proceedings of the 30th International Conference on International Conference on Machine Learning.

[55] Ganin Y., Lempitsky V. Unsupervised domain adaptation by backpropagation. Proceedings of the 32nd International Conference on International Conference on Machine Learning.

[56] Li Y., Tian X., Gong M., Liu Y., Liu T., Zhang K., Tao D. (2018). Deep domain generalization via conditional invariant adversarial networks. Computer Vision—ECCV 2018, Proceedings of the 15th European Conference, Munich, Germany, 8–14 September 2018.

[57] Shao R., Lan X., Li J., Yuen P.C. Multi-adversarial discriminative deep domain generalization for face presentation attack detection. Proceedings of the IEEE/CVF Conference on Computer Vision and Pattern Recognition.

[58] Jia Y., Zhang J., Shan S., Chen X. Single-side domain generalization for face anti-spoofing. Proceedings of the IEEE/CVF Conference on Computer Vision and Pattern Recognition.

[59] Björnson E., Sanguinetti L., Wymeersch H., Hoydis J., Marzetta T.L. (2019). Massive MIMO is a reality—What is next?: Five promising research directions for antenna arrays. Digit. Signal Process..

[60] Alrabeiah M., Zhang Y., Alkhateeb A. (2022). Neural Networks Based Beam Codebooks: Learning mmWave Massive MIMO Beams That Adapt to Deployment and Hardware. IEEE Trans. Commun..

[61] Di Renzo M., Zappone A., Debbah M., Alouini M.S., Yuen C., De Rosny J., Tretyakov S. (2020). Smart radio environments empowered by reconfigurable intelligent surfaces: How it works, state of research, and the road ahead. IEEE J. Sel. Areas Commun..

[62] Di Renzo M., Danufane F.H., Xi X., De Rosny J., Tretyakov S. Analytical modeling of the path-loss for reconfigurable intelligent surfaces—Anomalous mirror or scatterer?. Proceedings of the 2020 IEEE 21st International Workshop on Signal Processing Advances in Wireless Communications (SPAWC).

[63] Kammoun A., Chaaban A., Debbah M., Alouini M.S. (2020). Asymptotic max-min SINR analysis of reconfigurable intelligent surface assisted MISO systems. IEEE Trans. Wirel. Commun..

[64] Huang C., Zappone A., Alexandropoulos G.C., Debbah M., Yuen C. (2019). Reconfigurable intelligent surfaces for energy efficiency in wireless communication. IEEE Trans. Wirel. Commun..

[65] Huang C., Alexandropoulos G.C., Zappone A., Debbah M., Yuen C. Energy efficient multi-user MISO communication using low resolution large intelligent surfaces. Proceedings of the 2018 IEEE Globecom Workshops (GC Wkshps).

[66] Pei X., Yin H., Tan L., Cao L., Li Z., Wang K., Zhang K., Björnson E. (2021). RIS-aided wireless communications: Prototyping, adaptive beamforming, and indoor/outdoor field trials. IEEE Trans. Commun..

[67] Dai L., Wang B., Wang M., Yang X., Tan J., Bi S., Xu S., Yang F., Chen Z., Di Renzo M. (2020). Reconfigurable intelligent surface-based wireless communications: Antenna design, prototyping, and experimental results. IEEE Access.

[68] Méndez-Rial R., Rusu C., González-Prelcic N., Alkhateeb A., Heath R.W. (2016). Hybrid MIMO architectures for millimeter wave communications: Phase shifters or switches?. IEEE Access.

[69] Hemadeh I.A., Satyanarayana K., El-Hajjar M., Hanzo L. (2017). Millimeter-wave communications: Physical channel models, design considerations, antenna constructions, and link-budget. IEEE Commun. Surv. Tutor..

[70] Schneider T., Wiatrek A., Preußler S., Grigat M., Braun R.P. (2012). Link budget analysis for terahertz fixed wireless links. IEEE Trans. Terahertz Sci. Technol..

[71] Zyren J., Petrick A. (1998). Tutorial on Basic Link Budget Analysis.

[72] (2004). Zelst, van, A. MIMO OFDM for Wireless LANs. Ph.D. Thesis.

[73] Taha A., Alrabeiah M., Alkhateeb A. (2021). Enabling large intelligent surfaces with compressive sensing and deep learning. IEEE Access.

[74] Luo X., Meratnia N. A Geometric Deep Learning Framework for Accurate Indoor Localization. Proceedings of the 2022 IEEE 12th International Conference on Indoor Positioning and Indoor Navigation (IPIN).

[75] Wu Z., Pan S., Chen F., Long G., Zhang C., Philip S.Y. (2020). A comprehensive survey on graph neural networks. IEEE Trans. Neural Netw. Learn. Syst..

[76] Seybold J.S. (2005). Introduction to RF Propagation.

[77] Suh J., Kim C., Sung W., So J., Heo S.W. (2016). Construction of a generalized DFT codebook using channel-adaptive parameters. IEEE Commun. Lett..

[78] Henderson H.V., Pukelsheim F., Searle S.R. (1983). On the history of the Kronecker product. Linear Multilinear Algebra.

[79] Bronstein M.M., Bruna J., LeCun Y., Szlam A., Vandergheynst P. (2017). Geometric deep learning: Going beyond euclidean data. IEEE Signal Process. Mag..

[80] Shcherbakov M.V., Brebels A., Shcherbakova N.L., Tyukov A.P., Janovsky T.A., Kamaev V.A. (2013). A survey of forecast error measures. World Appl. Sci. J..

[81] Zhang Z., Wang H., Xu F., Jin Y.Q. (2017). Complex-valued convolutional neural network and its application in polarimetric SAR image classification. IEEE Trans. Geosci. Remote Sens..

[82] Cao Y., Wu Y., Zhang P., Liang W., Li M. (2019). Pixel-wise PolSAR image classification via a novel complex-valued deep fully convolutional network. Remote Sens..

[83] Alexandropoulos G.C., Vlachos E. A hardware architecture for reconfigurable intelligent surfaces with minimal active elements for explicit channel estimation. Proceedings of the ICASSP 2020-2020 IEEE International Conference on Acoustics, Speech and Signal Processing (ICASSP).

[84] Hamilton W., Ying Z., Leskovec J. Inductive representation learning on large graphs. Proceedings of the 31st International Conference on Neural Information Processing Systems.

[85] Gilmer J., Schoenholz S.S., Riley P.F., Vinyals O., Dahl G.E. Neural message passing for quantum chemistry. Proceedings of the 34th International Conference on Machine Learning.

[86] Maas A.L., Hannun A.Y., Ng A.Y. (2013). Rectifier nonlinearities improve neural network acoustic models. Proc. Icml.

[87] Nazarov I., Burnaev E. Bayesian Sparsification of Deep C-valued Networks. Proceedings of the 37th International Conference on Machine Learning.

[88] Arjovsky M., Shah A., Bengio Y. Unitary evolution recurrent neural networks. Proceedings of the 33rd International Conference on International Conference on Machine Learning.

[89] Venkateswaran V., van der Veen A.J. (2010). Analog beamforming in MIMO communications with phase shift networks and online channel estimation. IEEE Trans. Signal Process..

[90] Grant I.S., Phillips W.R. (2013). Electromagnetism.

[91] Hirose A. (2003). Complex-Valued Neural Networks: Theories and Applications.

[92] Barrachina J.A., Ren C., Morisseau C., Vieillard G., Ovarlez J.P. Complex-valued vs. real-valued neural networks for classification perspectives: An example on non-circular data. Proceedings of the ICASSP 2021-2021 IEEE International Conference on Acoustics, Speech and Signal Processing (ICASSP).

[93] Li Y., Luo Y., Yang G. (2017). 12-port 5G massive MIMO antenna array in sub-6GHz mobile handset for LTE bands 42/43/46 applications. IEEE Access.

[94] Pang J., Li Z., Kubozoe R., Luo X., Wu R., Wang Y., You D., Fadila A.A., Saengchan R., Nakamura T. 21.1 a 28GHz CMOS phased-array beamformer utilizing neutralized bi-directional technique supporting dual-polarized MIMO for 5G NR. Proceedings of the 2019 IEEE International Solid-State Circuits Conference-(ISSCC).

[95] Pauluzzi D.R., Beaulieu N.C. (2000). A comparison of SNR estimation techniques for the AWGN channel. IEEE Trans. Commun..

[96] Bonani F., Guerrieri S.D., Ghione G. (2003). Physics-based simulation techniques for small-and large-signal device noise analysis in RF applications. IEEE Trans. Electron Devices.

[97] Jiang Y., Li K., Gao J., Harada H. Antenna space diversity and polarization mismatch in wideband 60 GHz-Millimeter-wave wireless system. Proceedings of the 2009 IEEE 20th International Symposium on Personal, Indoor and Mobile Radio Communications.

[98] Dietrich C.B., Dietze K., Nealy J.R., Stutzman W.L. (2001). Spatial, polarization, and pattern diversity for wireless handheld terminals. IEEE Trans. Antennas Propag..

[99] Kwon S.C., Stüber G.L. (2014). Polarization division multiple access on NLoS wide-band wireless fading channels. IEEE Trans. Wirel. Commun..

[100] Tse D., Viswanath P. (2005). Fundamentals of Wireless Communication.

[101] Paszke A., Gross S., Massa F., Lerer A., Bradbury J., Chanan G., Killeen T., Lin Z., Gimelshein N., Antiga L. Pytorch: An imperative style, high-performance deep learning library. Proceedings of the 33rd International Conference on Neural Information Processing Systems.

[102] Wang M., Zheng D., Ye Z., Gan Q., Li M., Song X., Zhou J., Ma C., Yu L., Gai Y. (2019). Deep Graph Library: A Graph-Centric, Highly-Performant Package for Graph Neural Networks. arXiv.

[103] Kingma D.P., Ba J. Adam: A Method for Stochastic Optimization. Proceedings of the 3rd International Conference on Learning Representations, ICLR 2015.

